# Alterations in energy balance from an exercise intervention with *ad libitum* food intake

**DOI:** 10.1017/jns.2015.36

**Published:** 2016-03-09

**Authors:** Katarina Melzer, Anne Renaud, Stefanie Zurbuchen, Céline Tschopp, Jan Lehmann, Davide Malatesta, Nicole Ruch, Yves Schutz, Bengt Kayser, Urs Mäder

**Affiliations:** 1 Swiss Federal Institute of Sport, Magglingen, Switzerland; 2 Institute of Human Movement Sciences and Sport, Swiss Federal Institute of Technology, Zurich, Switzerland; 3 Faculty of Science, University of Freiburg, Freiburg, Switzerland; 4 Faculty of Biology and Medicine, Institute of Sports Sciences, University of Lausanne, Lausanne, Switzerland; 5 Department of Physiology, Faculty of Biology and Medicine, University of Lausanne, Lausanne, Switzerland; 6 Faculty of Biology and Medicine, Department of Physiology, University of Lausanne, Lausanne, Switzerland; 7 Integrative Cardiovascular and Metabolic Physiology, University of Freiburg, Freiburg, Switzerland

**Keywords:** Energy balance, Energy expenditure, *Ad libitum* food intake, Exercise intervention, Body composition, AEE, activity energy expenditure, ATM, adipose tissue mass, EE, energy expenditure, EI, energy intake, estV_O2max_, estimated maximal cardiorespiratory fitness, HR, heart rate, LTM, lean tissue mass, MET, metabolic equivalent, PA, physical activity, PAL, physical activity level, REE, resting energy expenditure, TEE, total energy expenditure, V_CO2_, carbon dioxide production, V_O2_, oxygen consumption

## Abstract

Better understanding is needed regarding the effects of exercise alone, without any imposed dietary regimens, as a single tool for body-weight regulation. Thus, we evaluated the effects of an 8-week increase in activity energy expenditure (AEE) on *ad libitum* energy intake (EI), body mass and composition in healthy participants with baseline physical activity levels (PAL) in line with international recommendations. Forty-six male adults (BMI = 19·7–29·3 kg/m^2^) participated in an intervention group, and ten (BMI = 21·0–28·4 kg/m^2^) in a control group. Anthropometric measures, cardiorespiratory fitness, EI, AEE and exercise intensity were recorded at baseline and during the 1st, 5th and 8th intervention weeks, and movement was recorded throughout. Body composition was measured at the beginning and at the end of the study, and resting energy expenditure was measured after the study. The intervention group increased PAL from 1·74 (se 0·03) to 1·93 (se 0·03) (*P* < 0·0001) and cardiorespiratory fitness from 41·4 (se 0·9) to 45·7 (se 1·1) ml O_2_/kg per min (*P* = 0·001) while decreasing body mass (−1·36 (se 0·2) kg; *P* = 0·001) through adipose tissue mass loss (ATM) (−1·61 (se 0·2) kg; *P* = 0·0001) compared with baseline. The control group did not show any significant changes in activity, body mass or ATM. EI was unchanged in both groups. The results indicate that in normal-weight and overweight men, increasing PAL from 1·7 to 1·9 while keeping EI *ad libitum* over an 8-week period produces a prolonged negative energy balance. Replication using a longer period (and/or more intense increase in PAL) is needed to investigate if and at what body composition the increase in AEE is met by an equivalent increase in EI.

Energy balance is the state in which energy expenditure (EE) equals metabolisable energy intake (EI) so that the overall energy content of the body remains stable^(^^1^^)^. A negative energy balance occurs when EE is greater than EI, and a positive energy balance occurs when EI is greater than EE. The extent to which exercise changes energy balance depends on the extent to which the increase in EE due to exercise is balanced by a compensatory increase in EI.

The intuitive common-sense view is that an increase in activity EE (AEE) is followed by an equivalent increase in EI^(^^2^^)^, which raises the question of whether exercise alone, without any imposed dietary regimens, could be prescribed as a single tool for body-weight regulation.

Some studies indicate that short-term exercise does not have the same effect on food intake as long-term exercise. Studies investigating the effects of exercise on *ad libitum* EI suggest a rather loose coupling between EI and EE in both normal-weight and obese individuals during short-term exercise interventions lasting from 1 or more hours to 2 or more weeks^(^^3^^–^^15^^)^. It was suggested that mobilisation of the fuels in the blood during exercise plays a role in the inhibition of food intake^(^^16^^)^. It was also found that exercise is accompanied by an increase in the release of glucagon, which in combination with other satiety factors such as cholecystokinin, cytokines and serotonin, contribute to suppressing food intake^(^^16^^,^^17^^)^. Exercise-induced increases in core temperature, blood lactate levels and TNF were also cited as possible mechanisms inducing hunger suppression^(^^18^^)^. All these mechanisms were proposed to explain the exercise-induced suppression of EI, but the exact mechanisms for this phenomenon remain largely unknown.

Although an increase of EE is not followed by an equivalent increase of EI in the short term^(^^19^^)^, the correlation becomes positive over the course of approximately 1 to 2 weeks or more, depending on the participant's body composition^(^^11^^,^^20^^)^. The speed at which EI starts balancing increased EE may differ between lean and obese participants. In lean active individuals with daily physical activity levels (PAL) around 1·7 and higher, energy balance is achieved by spontaneous adjustments of EI to match EE, yielding relative stability of the body's energy stores^(^^20^^)^. Several studies suggest that an increase in PAL is less likely to be followed by a corresponding increase in EI in obese individuals than in lean individuals^(^^21^^–^^25^^)^. Many physiological and psychological factors may influence the observed absence of a compensatory increase in EI in obese participants^(^^26^^)^. One explanation is that fat mass may act as an energy buffer^(^^3^^)^ and fully compensatory responses in EI to altered levels of AEE might not begin until the excess adipose tissue is expended^(^^26^^)^, but this hypothesis has not been directly tested.

The aim of the present study was to evaluate the effects of an 8-week exercise-induced increase of EE on *ad libitum* EI, body mass and body composition changes in normal-weight and overweight male participants with baseline PAL in line with international recommendations for the health-enhancing effects of physical activity (PA), i.e. at least 30 min of moderate intensity at least 5 d a week^(^^27^^,^^28^^)^. We hypothesised that increasing PA to regularly higher levels would increase *ad libitum* food intake only once excess body fat has been depleted.

## Experimental methods

### Participants

A total of sixty normal-weight and overweight healthy male adults (BMI = 19·7–29·3 kg/m^2^) were recruited in an intervention group, and fifteen participants (BMI = 21·0–28·4 kg/m^2^) were included in a control group. The sample size of the intervention group was calculated *a priori* (G*Power software, v. 3.1.9.2) using a *t* test for dependent samples calculation (α-error 0·05; power (1 – β) 0·80; effect size 0·5). We obtained a sample size of twenty-seven, which was rounded to thirty participants. Assuming a dropout rate of around 50 %, as reported in the literature^(^^29^^)^, we recruited a total of sixty participants. The participants were recruited in a non-randomised allocation manner, recruiting the intervention group first, followed by fifteen participants in the control group for the sake of data comparison and detection of possible significant differences between the groups. Participants were healthy individuals who engaged in at least 30 min of moderate intensity PA at least 5 d a week. They were non-smokers, had no known history of cardiovascular/metabolic disease, had stable body weight (<2·0 kg body-weight change in the last 6 months) and were not dieting or taking medications. All participants provided written informed consent to participate. The ethical committee of the Canton of Bern approved the study.

Out of sixty participants in the intervention group, fourteen dropped out: six participants dropped out due to individual work pressure that was incompatible with full participation in the intervention, two fell ill, two were injured, and there were technical problems in activity data recordings for four participants. In the control group, data analyses were excluded for five participants: three due to technical problems in activity data recordings and two due to uncompleted dietary data. The baseline participant characteristics did not differ between the intervention group and the control group (*P* > 0·05) (Table 1).

**Table 1.** Participants’ characteristics at baseline* (Mean values with their standard errors)

	Intervention (*n* 46)		Control (*n* 10)	
	Mean	se	Mean	se
Age (years)	28	1·0	29	2·4
Weight (kg)	81·8	1·1	79·6	3·6
Height (cm)	180·0	1·1	182·5	1·8
BMI (kg/m^2^)	25·3	0·3	23·9	1·1
estV_O2max_ (ml O_2_/kg per min)	41·4	0·9	37·8	1·2
estV_O2max_ (litres/min)	3·4	0·1	3·0	0·1
Lean tissue mass (kg)	58·1	0·8	57·5	1·9
Adipose tissue mass (kg)	20·7	0·8	19·2	2·1

estV_O2max_, estimation of cardiorespiratory fitness.

* There were no significant differences between the groups.

### Study design

**Fig. 1.**
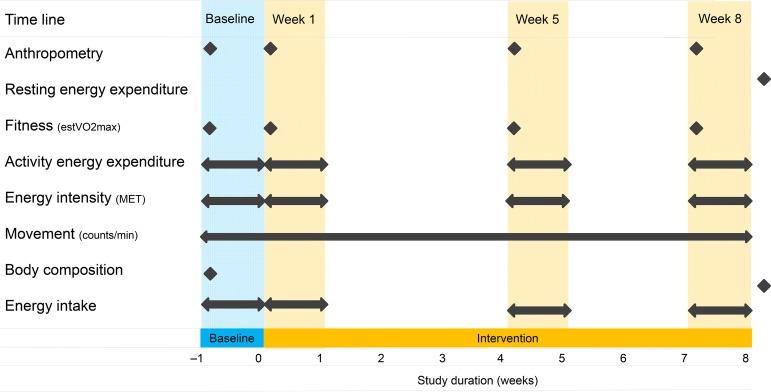
Schematic representation of the intervention group (*n* 46) study protocol. estV_O2max_, estimation of cardiorespiratory fitness; MET, metabolic equivalent of task.

The participants were recruited by advertisement, and they were kept naive as to the exact rationale of the study so that motivation for recruitment would not be connected with body-weight alteration. The declared purpose was to conduct metabolic studies. Beyond that, the true purpose of the study was not discussed with the participants, nor did they spontaneously inquire about it. The study consisted of a 1-week baseline period and an 8-week intervention period. During the baseline period, the participants were instructed to maintain their usual lifestyle routines, food intake and PA habits. After the baseline period, the intervention group was instructed to increase AEE by approximately 10·5 MJ/week or approximately 2092 kJ/d on 5 d/week on a moderate to vigorous PAL for the duration of the 8-week intervention period. The control group was instructed to maintain their usual lifestyle routines, food intake and PA habits throughout the entire study period. The Compendium of Physical Activity^(^^30^^)^ was used to provide participants in the intervention group with examples of endurance exercise modes (jogging, cycling, walking, swimming, etc.) necessary to expend approximately 2092 kJ/d. Upon request, some exercise modes (jogging, walking) were also demonstrated practically (intensity and duration) by a professional PA instructor. Anthropometric data, cardiovascular fitness, AEE, energy intensity and EI data were collected during the baseline period and during the 1st, 5th and 8th weeks of the intervention period in the intervention group. Movement (counts/min) during waking hours was recorded with accelerometers throughout the entire study. Body composition was measured at the beginning and at the end of the study, and resting EE (REE) was measured after the study. All the measurements were also repeated in the control group, except for the AEE and dietary intake during the 1st week of the intervention period, which were not carried out due to the limited financial and human resources available for the study. Before and after each measurement time point, the PA instructor organised regular meetings with the participants, discussed the activities performed and encouraged the maintenance of the prescribed PAL. All data were collected at the Swiss Olympic Medical Centre, Magglingen, Switzerland, using the methods described below. Fig. 1 provides a schematic representation of the intervention group study protocol.

### Anthropometric data

Participants arrived at the medical centre in the morning in a fasted state. Body weight was measured to the nearest 0·01 kg using a calibrated beam scale (Seca Ltd), and body height was measured to the nearest 0·01 cm using a height rod (Seca Ltd), with participants in underwear and without shoes.

### Body composition

Body composition was assessed at the beginning and end of the study using a Lunar iDXA (GE Healthcare). The participants wore underwear, and all metal artefacts were removed. Participants’ body composition was measured in the morning in a fasted state. On the day before the measurements, participants were instructed to refrain from PA and to eat an identical last meal before an overnight fast. Participants voided before each scan. During the measurements, participants were instructed to lay supine on the scanning table with their ankles fixed using supports. Participants’ arms were positioned to the side with their palms flat on the table. Participants were required to remain still. Whole-body scans were performed according to the manufacturer's instructions, and adipose tissue mass (ATM), lean tissue mass (LTM) and bone mineral content were calculated (enCore software v. 11.10; GE Healthcare). The machine's calibration was checked and passed on a daily basis before each scanning session using a calibration phantom. Total body composition estimates with the Lunar iDXA have been reported to be excellent in other studies^(^^31^^,^^32^^)^.

### Resting energy expenditure

REE was assessed by indirect calorimetry using a metabolic cart (MOXUS Metabolic System; AEI Technologies Inc.). Calibration of the gas analysers and flow measurement module was carried out before each measurement according to the manufacturer's instructions. The participants were instructed to arrive at the research unit in the morning after an overnight fast, avoiding any strenuous physical effort. After acclimatising and relaxing on a bed for 30 min, a ventilated hood (Canopy System Option, AEI Technologies, Inc.) was placed over their heads and the measurements started. Oxygen consumption (V_O2_) and carbon dioxide production (V_CO2_) were measured for 30 min with the participants in a supine position and completely at rest in a quiet and thermoneutral environment (20–22°C). The first 5 min of data were eliminated as an acclimatisation artefact. From the remaining 25 min, segments of a minimum ten consecutive 1-min measures with <10 % CV in V_O2_ and V_CO2_ were considered as steady-state. V_O2_ and V_CO2_ were then used to calculate REE using the abbreviated Weir equation^(^^33^^)^. Due to the malfunctioning of the metabolic cart at one study point, we succeeded in obtaining measured REE values only for forty-two participants out of the total number recruited. Using a statistical regression model, an equation for estimating REE that best fit the measured data was developed: (*R* 0·74): REE = 911·325 – (5·997 × age (year)) + (14·628 × LTM (kg)) + (8·903 × ATM (kg)). This equation was used to estimate REE for all participants.

### Energy expenditure and cardiovascular fitness

AEE was estimated analysing full day (24 h) recordings of heart rate (HR) and body movement with a 15-s averaging epoch setting during the baseline week and in the 1st, 5th and 8th weeks of the intervention period. A non-invasive, lightweight (10 g), waterproof combined HR and movement sensor (accelerometer) device (Actiheart v. 4.0.109; CamNtech Ltd) was clipped onto two ECG electrodes (3 M Red Dot Electrode 3560) on the left thorax just below the apex of the sternum^(^^34^^)^. Sleeping HR was measured as the highest value of the thirty lowest minute-by-minute HR readings during a 24-h day. If the epoch was 15 s, then 120 readings were used.

The device was calibrated for each use (baseline and the 1st, 5th and 8th weeks of the intervention period) for each participant using a standard step test. The step test is an in-built function of the Actiheart software that is designed to estimate the maximal cardiovascular fitness (estV_O2max_) of an individual (for detailed explanations, see Brage *et al*.^(^^35^^,^^36^^)^). The reliability and validity of the device have been established elsewhere^(^^35^^,^^37^^)^. The Actiheart has been proven to provide an accurate estimate against indirect calorimetry during a wide range of activities in men and women (from low to moderate and high activities) in both laboratory^(^^38^^)^ and field settings^(^^39^^)^. The mean errors of the individually calibrated estimates were shown to be 1·5 %^(^^36^^)^. Moreover, a recent study supported a good level of agreement between the individually calibrated accelerometer/HR model of the Actiheart (used for this study) and doubly labelled water for measuring AEE in lean and overweight men with varying fitness levels in free-living conditions^(^^40^^)^.

Total EE (TEE) was calculated as a sum of the AEE, REE and diet-induced thermogenesis (assumed at 10 % of TEE)^(^^36^^)^. PAL was calculated as TEE/REE.

### Energy intensity

The total intensity of activities was presented in metabolic equivalents (MET) and calculated as a multiple of REE. The MET values are presented in the following intervals: MET < 3 (sedentary to low PA); MET = 3–6 (moderate PA); MET > 6 (vigorous PA).

### Physical activity movement

PA movement was measured throughout the study over the entire baseline (7 d) and exercise intervention period (8 weeks) using accelerometers (ActiGraph GT3X). The ActiGraph is a small and light (3·8 × 3·7 × 1·8 cm^3^, 27 g) device programmed to record activity counts. The epoch interval used was set at 1 min, and output was expressed as mean daily counts per min. The device measured accelerations from 0·05 up to 2·5 ***g***. An electronic filter inside the accelerometer limited the device to a frequency of 0·25 to 2·5 Hz. The settings thus captured normal human motion but filtered out high-frequency vibrations from mechanical sources. For consistency, all participants wore the ActiGraph accelerometer on their right hips with the same elastic belt and adjustable buckle^(^^41^^,^^42^^)^. Participants were instructed to remove the device only at night and during swimming, showering or bathing. A valid day was defined as 10 or more hours of wear time^(^^43^^)^. Wear time was defined by subtracting non-wear time from 24 h. Non-wear time was defined as at least 60 consecutive min of zero counts, with allowance for 1 to 2 min of counts between 1 and 100^(^^43^^)^. The data were downloaded according to the manufacturer's specifications using software provided by the company (ActiLife v. 6.5.3; ActiGraph). Mean values of the vertical counts per min per valid day (count/min) were then calculated and used for further analyses. Validation of the device has been reported elsewhere^(^^44^^)^.

### Energy intake

Dietary patterns and alcohol consumption were monitored through 7-d dietary records. A certified nutritionist explained to each participant how to write down the type and amount of food eaten. Detailed descriptions of all foods and beverages consumed, including cooking methods and brand names, were recorded by the participants. The dietary intake data were analysed using EPISpro dietary assessment software (EPISpro, BLS 3.01; University of Hohenheim, Stuttgart, Germany). When food items were not found in the database (Swiss Food Composition Data v. 3.01; ETH Zurich & Federal Office of Public Health, Switzerland), they were broken down into their individual ingredients for analysis.

### Statistical analyses

Descriptive data for continuous variables are reported as means with their standard errors. All study measurements were normally distributed according to the Shapiro–Wilk test (*P* > 0·05). Evaluation variables were compared between different measurement time points or different groups at the same time point using a paired and unpaired *t* test. A one-way ANOVA (with repeated measures) was applied to compare means at three time points. An ANCOVA was also used when controlling for the effects of PAL. We considered a *P* value <0·05 to indicate a significant difference. SPSS software (SPSS, Inc., v. 22) was used for data description and statistical analysis.

## Results

In the intervention group, the AEE of 4·2 (se 0·2) MJ/d during the baseline period increased to 5·5 (se 0·2), 5·6 (se 0·2) and 5·3 (se 0·2) MJ/d in the 1st, 5th and 8th weeks of the intervention period, respectively. These values were all significantly different from the baseline (*P* = 0·0001). The AEE thus increased by 1·2 (se 0·3), 1·4 (se 0·3) and 1·1 (se 0·3) MJ/d, respectively, compared with the baseline values, with an average increase of 1·2 (se 0·2) MJ/d. The AEE of the 1st, 5th and 8th weeks of the intervention period were similar (*P* = 0·42). The control group did not differ in AEE compared with the intervention group during the baseline period (3·6 (se 0·3) *v*. 4·2 (se 0·2) MJ/d, respectively; *P* = 0·11). Over the course of the intervention period, the control group did not show changes in mean AEE (4·0 (se 0·3) MJ/d) compared with the baseline values (*P* = 0·32).

The mean daily number of acceleration counts in the intervention group during the 1st, 5th and 8th intervention weeks was 302 (se 12) counts/min, and it did not significantly differ from the mean number of acceleration counts in the 2nd, 3rd, 4th, 6th and 7th intervention weeks (301 (se 12) counts/min; *P* = 0·92). The mean acceleration counts through the entire 8-week intervention period were significantly higher compared with the baseline values (302 (se 12) *v*. 226 (se 11) counts/min; *P* = 0·001). The mean acceleration counts during the intervention period of the control group did not change compared with their baseline values (244 (se 18) *v*. 236 (se 25) counts/min; *P* = 0·64, respectively).

Times (min/d) spent on sedentary to low (MET < 3), moderate (MET = 3–6) and vigorous (MET > 6) PA during the baseline period and the intervention period (5th and 8th intervention weeks) are presented in Table 2. The time spent on all three intensities was similar between the intervention and the control groups during the baseline period (all *P* > 0·05). During the intervention period, the intervention group decreased time spent in MET < 3 by an average of 34 (se 9) min/d (*P* < 0·05) and increased time spent in moderate (MET = 3–6) and vigorous activities (MET > 6) by an average of 18 (se 7) min/d (*P* < 0·05) and 14 (se 2) min/d (*P* < 0·05), respectively. The time spent in all intensities did not change during the intervention period in the control group (*P* > 0·05). The respective mean time spent on low, moderate and vigorous PA during the intervention period was significantly different between the control and intervention groups (all *P* < 0·05).

**Table 2.** Time (min/d) spent on sedentary to low (metabolic equivalent (MET) < 3), moderate (MET = 3–6) and vigorous (MET > 6) physical activity during the baseline and intervention periods (mean value of the 5th and 8th intervention week) (Mean values with their standard errors)

	Baseline period		Intervention period	
	Mean	se	Mean	se
MET < 3				
Intervention group	1342	6	1309*†	7
Control group	1369	8	1353	7
MET = 3–6				
Intervention group	89	5	107*†	6
Control group	64	7	78	7
MET > 6				
Intervention group	9	1	24*†	7
Control group	7	2	9	3

* Mean value was significantly different from that of the control group (*P* < 0·05).

† Mean value was significantly different from that of the baseline period (*P* < 0·05).

**Fig. 2.**
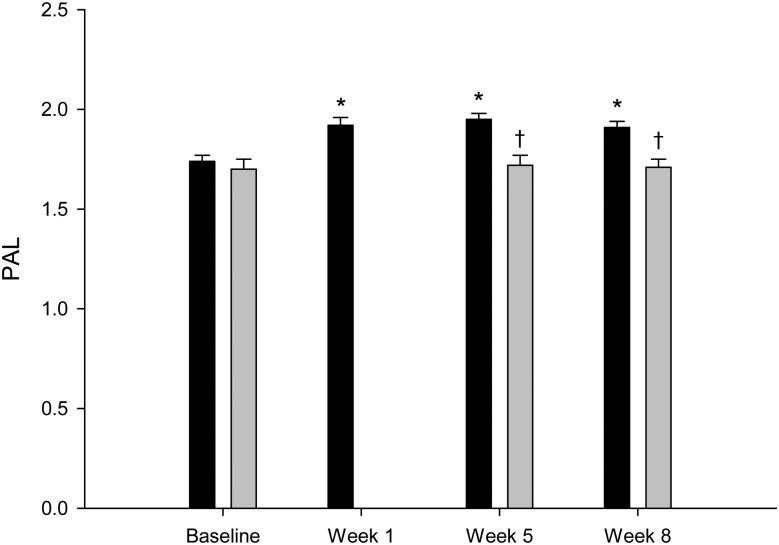
Physical activity level (PAL) in the intervention group (■; *n* 46) and in the control group (▒; *n* 10). Values are means, with standard errors represented by vertical bars. * Mean value was significantly different from that at baseline (*P* < 0·05). † Mean value was significantly different from that of the intervention group (*P* < 0·05).

In the intervention group, the PAL increased from 1·74 (se 0·03) during the 7-d baseline period to 1·92 (se 0·03), 1·95 (se 0·03) and 1·91 (se 0·03) during the 1st, 5th and 8th weeks of the intervention period, respectively, with an average value of 1·93 (se 0·03) (Fig. 2). All values were significantly different from the baseline values (*P* < 0·0001). The control group did not differ in PAL compared with the intervention group during the baseline period 1·66 (se 0·05) *v*. 1·74 (se 0·03); *P* = 0·15). The PAL of the control group during the 5th and 8th weeks of the intervention period was 1·72 (se 0·05) and 1·70 (se 0·04), respectively (*P* = 0·72). The mean PAL of the control group during the intervention period (1·71 (se 0·04)) was similar to the baseline value (*P* = 0·32). The mean PAL values (5th and 8th intervention weeks) between the control and intervention groups during the intervention period were significantly different (*P* = 0·002).

In the intervention group, estV_O2max_ increased from 41·4 (se 0·9) ml O_2_/kg per min during the baseline period to 45·7 (se 1·1) ml O_2_/kg per min at the end of the intervention period (*P* = 0·001) (3·38 (se 0·1) and 3·67 (se 0·1)  litres O_2_/min, respectively; *P* = 0·001). In the control group, estV_O2max_ values did not change significantly between the two study periods (37·8 (se 1·2) *v*. 40·8 (se 2·5) ml O_2_/kg per min; *P* = 0·22) (3·0 (se 0·1) and 3·2 (se 0·2) litres O_2_/min, respectively; *P* = 0·24).

**Fig. 3.**
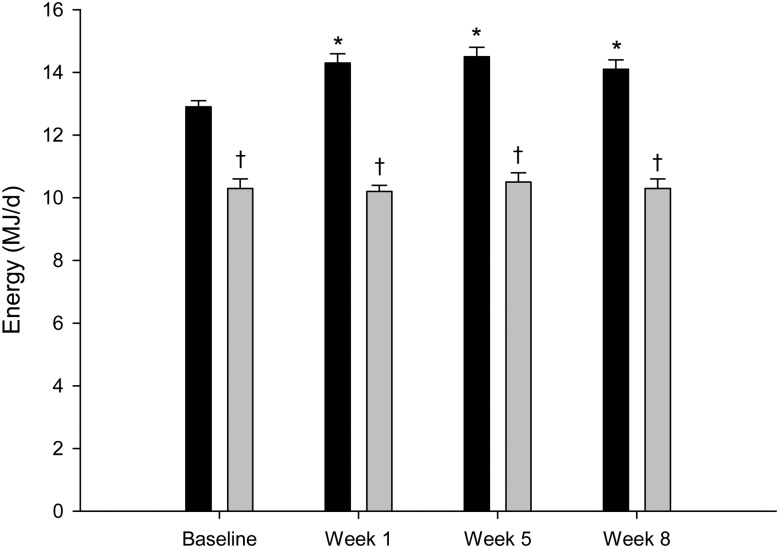
Total energy expenditure (TEE; ■) and energy intake (▒) in the intervention group (*n* 46). Values are means, with standard errors represented by vertical bars. * Mean value was significantly different from that at baseline (*P* < 0·05). † Mean value was significantly different from that for TEE (*P* < 0·05).

Fig. 3 shows EI and TEE of the intervention group. The baseline EI was 10·3 (se 0·3) MJ/d, and EI during the 1st, 5th and 8th intervention weeks was 10·2 (se 0·2), 10·5 (se 0·3) and 10·3 (se 0·3) MJ/d, respectively. All these values were not different from the baseline EI (*P* = 0·32, *P* = 0·35 and *P* = 0·14, respectively). The baseline TEE was 12·9 (se 0·2) MJ/d, and TEE during the 1st, 5th and 8th intervention weeks was 14·3 (se 0·3), 14·5 (se 0·3) and 14·1 (se 0·3) MJ/d, respectively. These values were all significantly different from the baseline (*P* = 0·0001). EI was significantly different from TEE at any measured study point (*P* < 0·05). The inter-individual variability of EI and TEE during the intervention period was 7·0–14·3 and 10·2–18·4 MJ/d, respectively.

The baseline EI of the control group was 9·7 (se 0·5) MJ/d. During the 5th and 8th intervention weeks, EI of the control group was 9·6 (se 0·3) and 9·3 (se 0·5) MJ/d, respectively. These results showed no significant difference compared with the baseline EI (*P* = 0·78 and *P* = 0·21, respectively). The baseline TEE of the control group was 12·1 (se 0·5) MJ/d, and TEE during the 5th and 8th intervention weeks was 12·6 (se 0·5) and 12·4 (se 0·5) MJ/d, respectively. These values were not significantly different from the baseline (*P* = 0·27 and *P* = 0·48, respectively). The inter-individual variability of EI and TEE during the intervention period was 7·9–11·1 and 10·8–14·7 MJ/d, respectively.

EI of the control and intervention groups was similar, both at baseline (*P* = 0·4) and during the 5th and 8th intervention weeks (*P* = 0·22 and *P* = 0·16, respectively). The baseline TEE of the control and intervention groups was similar (*P* = 0·11). However, during the 5th and 8th intervention weeks, TEE of the control and intervention groups was significantly different (*P* = 0·01 and *P* = 0·02, respectively).

**Fig. 4.**
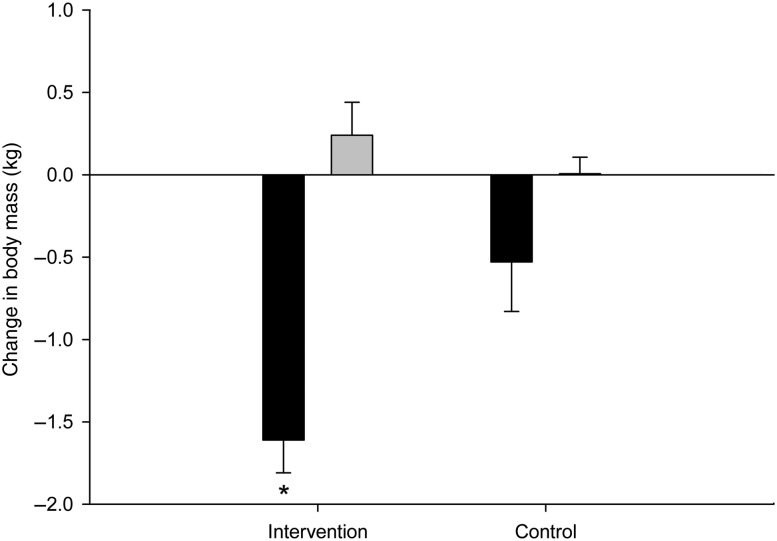
Adipose tissue mass (■) and lean tissue mass (▒) changes of the intervention (*n* 46) and control (*n* 10) groups after an 8-week exercise intervention. Values are means, with standard errors represented by vertical bars. * Mean value was significantly different from that of the control group (*P* < 0·05).

ATM and LTM changes from the beginning until the end of the study in the intervention and control groups are presented in Fig. 4. The range of body-weight change of the intervention group was wide (−6·2 to 2·7 kg), with thirty-eight participants (83 %) losing weight and eight participants (17 %) gaining weight. The BMI of the participants who gained weight was similar to the BMI of those who did not (25·7 (se 0·4) *v*. 25·2 (se 0·4) kg/m^2^, respectively; *P* = 0·56). The participants who gained weight had the same PAL as the participants who lost weight (1·90 (se 0·8) *v*. 1·94 (se 0·3), respectively; *P* = 0·65), with the same amount of time spent in sedentary, moderate or vigorous PA (1319 (se 16) *v*. 1308 (se 7) min/d, 99 (se 12) *v*. 107 (se 6) min/d, 22 (se 4) *v*. 25 (se 2) min/d; *P* = 0·52, *P* = 0·55 and *P* = 0·57, respectively). EI of the two groups was similar (10·5 (se 0·5) *v*. 10·3 (se 0·3) MJ/d, respectively; *P* = 0·76).

On average, the intervention group significantly lost body mass (−1·36 (se 0·2) kg; *P* = 0·001) and ATM (−1·61 (se 0·2) kg; *P* = 0·0001) compared with the baseline values, whereas LTM did not change significantly (0·24 (se 0·1) kg; *P* = 0·121). The control group did not show any significant changes in body mass (−0·6 (se 1·7) kg; *P* = 0·62), ATM (−0·53 (se 0·4) kg; *P* = 0·1) or LTM (−0·006 (se 0·2) kg; *P* = 0·9). The intervention group did not significantly decrease in body mass (*P* = 0·20) or significantly increase in LM (*P* = 0·50), but it did significantly decrease in ATM (*P* = 0·02) compared with the control group.

No significant difference was observed between overweight and normal-weight participants in terms of body mass, ATM and LTM. On average, overweight participants (BMI ≥ 25·0 kg/m^2^; *n* 27) lost 1·73 (se 0·4) kg while normal-weight participants (BMI < 25 kg/m^2^; *n* 19) lost 0·81 (se 0·3) kg body mass (*P* = 0·8). The ATM loss of overweight and normal-weight participants was 1·9 (se 0·3) and 1·2 (se 0·2) kg (*P* = 0·08), respectively. The LTM gain of overweight and normal-weight participants was 0·2 (se 0·2) and 0·3 (se 0·2) kg (*P* = 0·7), respectively.

When controlling for the effects of PAL in the intervention group, the overweight participants lost more body mass compared with the normal-weight (BMI < 25 kg/m^2^; *n* 19) participants (−1·73 (se 0·3) *v*. −0·81 (se 0·3) kg), but the difference was found to be non-significant (*P* = 0·08). EI between these groups was similar (*P* = 0·57).

## Discussion

The main important finding of the present study was that in normal-weight and overweight male participants largely meeting minimal recommendations for PA, an increase in PAL from 1·7 to 1·9 over an 8-week period did not result in significant changes in *ad libitum* EI. As a consequence, this was accompanied by a significant reduction of body mass through ATM loss.

Current guidelines recommend 30 min of moderate intensity activity 5 or more days per week for the general population to promote optimal health and prevent chronic diseases, 60 min/d of moderate intensity activity 5 or more days per week as a target level for preventing weight gain and 60–90 min of moderate intensity activity as a target level for preventing weight regain following significant weight loss and to enhance long-term weight control outcomes^(^^27^^,^^45^^–^^48^^)^. This consensus has been reached based predominantly on observational evidence^(^^33^^)^. One major limitation of the existing evidence is the lack of objective measurement of PA. Limited evidence indicates that a much higher dose of activity may be needed to prevent overweight and obesity and to avoid weight regain in previously overweight and obese individuals^(^^33^^)^.

Our participants were already active before starting the intervention period, engaging in PA for an average of 90 min/d at a moderate intensity level and around 10 min/d at a high intensity level, thus reaching an average baseline PAL of 1·7. This means that they were, already at baseline, largely compliant and much above the recommendations for minimal PA levels for the prevention of chronic disease. During the 8-week exercise intervention, PAL increased to an average of 1·9 (equivalent to around 2 h of moderate-to-high intensity activity per d). This increase of PAL did not only induce body mass loss during *ad libitum* food intake but also significantly improved a major protective factor, i.e. aerobic fitness level.

Although we would need to look at different PAL to be able to make recommendations on the PAL required for the prevention of body-weight gain (or regain), the findings of our study suggest a PAL of 1·9 as a reasonable level for body-weight gain prevention under *ad libitum* nutritional conditions.

According to the dietary reference intakes (DRI), the nutrient requirements of persons aged 19 to 50 years old are essentially equal and not influenced by ageing^(^^49^^)^. The participants in our study belonged to this age group (21 to 45 years), so their nutritional requirements were not influenced by age span. Additionally, we recruited only men in order to exclude the potential influence of sex in response to an imposed exercise intervention. While some studies showed no sex difference in body-weight response to exercise^(^^50^^)^, others suggested that women tend to ‘defend’ body fat against body mass loss due to increased PA under *ad libitum* food intake more effectively than men^(^^23^^,^^51^^,^^52^^)^. Finally, our inclusion criteria comprised both normal-weight and overweight participants. The body composition of individuals engaging in sports plays an important role in EI regulation and its changes over time. When engaging in moderate to intense PA regularly and on a long-term basis, lean and obese participants do not behave identically. Whereas lean participants show a tendency to balance the increased PAL by adapting their EI to reach a balance within a period of several days to several weeks, obese participants, probably due to their excess energy storage, do not show such a compensatory mechanism^(^^20^^)^. One possible explanation for this observation is that, in the obese state, fat mass may act as an energy buffer, and compensatory responses in intake to altered levels of exercise might not begin until excess energy stores drop below a certain level. Taking this into account, we have chosen to study normal-weight to overweight participants in order to detect possible alterations of EI due to increased PAL already during the 8-week exercise intervention period of our study. The larger excess body fat storage of obese participants would probably have required a significantly longer intervention period in order for their regulatory mechanism to trigger a spontaneous increase in EI once their energy stores dropped below a certain level.

As hypothesised, the 8-week exercise intervention did not progressively induce a reciprocal increase in EI. The overweight participants tended to lose more body weight than the normal-weight participants, despite having a similar PAL. However, the absence of a full compensatory effect in response to an exercise-induced energy deficit cannot be presumed to continue indefinitely. We would expect that, once the excess adipose tissue becomes significantly depleted, a regulatory mechanism would trigger a spontaneous increase in EI in order to match EE. To test this further, a similar study design over a longer period of time – or a study of similar duration but with a leaner study group – would be needed.

When examining the effects of exercise on body-weight regulation, it should also be taken into account that large inter-individual differences are often observed^(^^53^^)^. Examining only the overall group mean weight loss could lead to the inaccurate interpretation that all individuals experience the same effect of exercise. Our study also showed that exercise interventions produce large inter-individual variability in body weight. Furthermore, the participants who gained weight during the intervention period had a similar PAL and similar time spent on low, moderate or vigorous activities compared with those who lost weight. According to the dietary records of the participants who gained weight, their EI was similar to that of the individuals who lost weight, indicating possible larger EI under-reporting of those who gained weight.

To assess the effect of exercise on energy balance regulation, studies accurately and precisely measuring both EI and EE are needed. In the past, the lack of methods enabling precise and accurate estimates of AEE in a free-living environment for prolonged periods of time were responsible for the greater use of EI estimates as a proxy of EE, assuming EI = EE. However, in 1985, the joint FAO/WHO/UNU Expert Consultation suggested abandoning this approach and concentrating on measuring EE rather than EI to assess energy requirements^(^^54^^)^. In free-living humans, the accuracy of EI estimates is questionable and extremely uncertain^(^^55^^)^. Underestimations of EI up to 20 %, particularly in obese individuals^(^^55^^,^^56^^)^, have been observed. The underestimation in EI reporting is evident in the present study as well, starting from the baseline period, where one would assume EI would be similar to TEE, given that the participants reported stable body mass upon recruitment.

In order to enhance the accuracy of dietary assessment methods, future studies should explore the use of alternative techniques for EI measurement. The doubly labelled water method represents the ‘gold standard’ for estimating EE and validating EI measurements, although it is rather costly^(^^57^^)^. It has been recently suggested that wearable camera-assisted methods could reduce measurement error by revealing unreported foods and misreporting errors^(^^58^^)^. This method may have a smaller measurement error for EI (7 %)^(^^59^^)^ and may increase the accuracy of self-reported EI by 6–18 % compared with traditional methods^(^^58^^,^^60^^)^.

The causal relationship between sufficient regular PA and numerous aspects of health is beyond doubt, and physical inactivity was recently labelled ‘the biggest public health problem of the 21st century‘^(^^61^^)^. Activity promotion interventions have shown limited effectiveness for the general population, with an estimated 45 % dropout rate among individuals who initiate exercise programmes^(^^29^^)^. Our study participants were not sedentary and were meeting minimal recommendations for PA prior to entering the study. Even so, the dropout rate of 23 % in the intervention group is illustrative of the difficulties (lack of time, discipline and/or motivation) in integrating higher PAL into everyday life as this would entail a change in lifestyle that goes against the idea fostered by ‘modern’ life and its motorised and food-abundant surroundings.

Evidence from existing randomised controlled trials is relatively consistent with regard to the role of exercise alone in weight loss^(^^62^^)^. In studies where exercise results in only modest weight loss, the level of exercise prescribed was relatively low and presumably induced an energy deficit smaller than that generally recommended for weight loss by energy restriction^(^^63^^,^^64^^)^. The resulting weight loss findings from those studies are therefore consistent with the amount of exercise prescribed^(^^65^^)^. Limitations in existing studies include poor adherence to the prescribed PA, variability in the amount of exercise prescribed and the limited duration of the exercise interventions^(^^62^^)^. Few studies have addressed the role of activity in weight-loss maintenance by providing a long-term, sustained activity intervention, and there is a need for well-designed, prospective, randomised trials to assess such regimens.

### Limitations and strengths of the study

The present study should be interpreted in light of its limitations. First, the limited financial and human resources available for this study did not allow us to perform a randomised controlled trial by recruiting the same number of participants for the control and intervention groups. Therefore, the participants were recruited in a non-randomised allocation manner by recruiting the intervention group first, followed by fifteen participants in the control group, for the sake of data comparison and detection of possible significant differences between the groups. Although a non-randomised design could be susceptible to bias, we were careful to recruit the control group with the same participants’ characteristics as the intervention group, to present information on the study in the same way to both groups and to interpret the results in line with the study's non-randomised design. Second, the resources and equipment available at the relevant time did not allow us to measure REE both at the beginning and at the end of the study. Although the absence of these measures did not allow us to precisely measure possible changes in REE following the intervention period, we would not expect that any such change would substantially influence the overall results given that the amount of LTM was unchanged before *v*. after the intervention period.

Another limitation of the study lies in the fact that, due to limited financial and human resources, the AEE and dietary intake data of the control group could not be collected during the first week of the intervention period. Collecting these data would have allowed us to perform additional statistical analyses and further tease out the effect of time and/or condition on the outcomes. Additionally, we recruited only men to exclude potential influences related to sex in response to an imposed exercise intervention. Nevertheless, it would be interesting to perform a similar follow-up study on female participants as well, with the intention to examine possible differences in body-weight response to exercise. Finally, our study did not focus on analysing subjective or appetite hormone measures or eating behaviour, which potentially would have further contributed to the interpretation of our findings. Further studies are needed to find the mechanisms responsible for alterations in appetite and eating behaviour with exercise.

Our free-living design precluded any precise measurement of EI. Our participant-reported EI levels are probably biased by under-reporting, as can be seen from the energy gap at baseline (Fig. 3). Even though we cannot exclude that the extent of under-reporting changed during the following 8 weeks, the invariant EI over the course of the 8 weeks would suggest that the participants simply continued their habitual EI throughout the entire intervention period. We therefore assumed that EI did not change for the duration of the study. Since our control group did not show any changes over time, we are confident that the observations in the intervention group reflect the physiology of increased EE on body composition while *ad libitum* EI remained constant.

A strength of our study is that we tried to fill a gap in current knowledge related to the effects of an increase in AEE on *ad libitum* EI according to the recommendations of Donnelly *et al.*^(^^66^^)^, who systematically reviewed all studies published between 1990 and 2013 on this topic. They highlighted a need for adequately powered trials of sufficient duration that prescribe and measure exercise EE across the duration of the study and evaluate and compare the levels of exercise for weight management currently recommended by governmental agencies or professional organisations.

A novelty of the present study is that it focused not on obese but rather on normal-to-overweight participants. Our results show that the prevailing common-sense view, namely that an increase of AEE is followed by an equivalent increase in EI, does not hold true, even for participants who are, according to the BMI cut-off specifications, categorised as normal-weight to overweight. However, the observed absence of a compensatory effect of EI in response to an exercise-induced energy deficit cannot be presumed to continue indefinitely; otherwise, a considerable loss of body mass would occur. At some stage, a regulatory mechanism must trigger an increase in EI in order to match increased EE. The percentage of body fat, as a trigger point when EI starts balancing the increased exercise-induced EE, merits further investigation.

### Conclusion

The results of the present study indicate that in normal-weight and overweight men, increasing PAL from 1·7 to 1·9 during *ad libitum* EI over an 8-week period produces a prolonged negative energy balance. Replication of the study using a more prolonged (and/or greater increase in PAL) is needed to investigate if and at what body composition the increase in AEE is eventually met by an increase in EI.
